# Translating Trial to Treatment: A Scoping Review of Resmetirom First Year in Real-World Use for Moderate to Advanced Fibrosis in Metabolic Dysfunction-Associated Steatohepatitis

**DOI:** 10.7759/cureus.90268

**Published:** 2025-08-17

**Authors:** Kanika Vats, Mohammad Mazhar Alam

**Affiliations:** 1 Research and Innovation, TASNEEF (Emirates Classification Society), Abu Dhabi, ARE; 2 Laboratory, Disease Prevention Screening Center, Abu Dhabi, ARE

**Keywords:** cost of therapy, hepatic steatosis (masld), liver cirrhosis. fibrosis, metabolic dysfunction-associated steatohepatitis (mash), nonalcoholic fatty liver disease (nafld), non-alcoholic steatohepatitis (nash), post-marketing surveillance, real-world evidence, resmetirom, thyroid hormone receptor beta

## Abstract

Metabolic dysfunction-associated steatohepatitis (MASH), formally known as nonalcoholic steatohepatitis (NASH), a progressive subtype of nonalcoholic fatty liver disease (NAFLD), remains a major global contributor to liver-related morbidity and mortality. In early 2024, a new therapeutic option, Resmetirom, a liver-directed thyroid hormone receptor beta (THR-β) agonist, became available in the United States for individuals with MASH having moderate to advanced liver fibrosis (F2-F3), offering a targeted approach to treatment.

This review aims to explore and consolidate real-world experience, clinical insights, regulatory developments, and implementation practices related to the use of Resmetirom within its first year of availability.

This scoping review was conducted using a structured methodology aligned with the Preferred Reporting Items for Systematic reviews and Meta-Analyses extension for scoping reviews (PRISMA-ScR) guidelines. Relevant literature was identified through systematic searches in PubMed, Google Scholar, and clinical trial registries, using Boolean operators (AND). Search specificity was tested during strategy development, and studies published between April 2024 and May 2025 were included based on PICOS (Population, Intervention, Comparison, Outcomes, and Study Designs) criteria, focusing on adults with noncirrhotic MASH and F2-F3 fibrosis treated with Resmetirom. Study designs included clinical trials, observational studies, post-market reports, and real-world evidence. Supplementary data were extracted from Food and Drug Administration (FDA) and European Medicines Agency (EMA) announcements and proceedings from major liver congresses - American Association for the Study of Liver Diseases (AASLD) and European Association for the Study of the Liver (EASL). The findings from the 20 studies included in this review were described using a narrative approach and grouped according to key themes.

Key clinical trials and real-world data from the United States show that Resmetirom is effective in treating MASH, lowering liver fat and improving liver scarring in patients with moderate to advanced fibrosis (F2-F3). It also appears to be safe and well-tolerated, with good early adherence. Noninvasive tests to diagnose and monitor the disease are being used more often, but relying mainly on FIB-4 might miss some advanced cases. However, access to Resmetirom remains a challenge due to its high cost, insurance barriers, and limited evidence from global or high-risk populations. These issues highlight the need for fairer access and more post-approval studies to ensure broader use and understanding of the drug.

In its first year of post-approval use, Resmetirom has shown consistent efficacy, safety, and quality-of-life benefits for patients with MASH and fibrosis. However, real-world adoption remains limited by high costs, complex access pathways, underrepresentation of high-risk populations, and a lack of global data. This review highlights existing evidence gaps and stresses the importance of global validation, improved diagnostic tools, and fair access to ensure Resmetirom can achieve its full clinical potential.

## Introduction and background

Metabolic dysfunction-associated steatohepatitis (MASH), formally known as nonalcoholic steatohepatitis (NASH) [1,2], a progressive form of nonalcoholic fatty liver disease (NAFLD) [3], with rising global prevalence, from 25.26% during 1990-2006 to 38% in the period between 2016 and 2019, based on clinical indication such as elevated liver enzymes, makes it a major contributor to liver-related morbidity and mortality [4,5]. For the purpose of this article, the terms MASH and NASH are used interchangeably, as they appear in the source references.

NASH is marked by hepatic fat accumulation, inflammation, and progressive fibrosis, and in many cases, it can lead to cirrhosis, hepatocellular carcinoma (HCC), and ultimately liver failure [6-8]. Until recently, management relied solely on lifestyle interventions such as diet and physical activity [9]. While beneficial, these are often insufficient for patients with moderate to advanced fibrosis (F2-F3), where the risk of disease progression is more imminent.

A major therapeutic breakthrough occurred in March 2024, when the U.S. Food and Drug Administration (FDA) approved Resmetirom (brand name Rezdiffra), a selective thyroid hormone receptor-β (THR-β) agonist, for the treatment of adults with noncirrhotic NASH and moderate to advanced fibrosis [10]. The approval was based on promising results from the Phase III Madrigal's Efficacy and Safety Trial Evaluating Resmetirom in NASH (MAESTRO-NASH) trial, which demonstrated that Resmetirom significantly improved both NASH resolution and liver fibrosis compared to placebo [11,12]. In addition to histological benefits, treatment with Resmetirom was associated with meaningful reductions in hepatic fat content, liver stiffness, serum lipids, and transaminases [13,14].

Resmetirom demonstrated a favorable safety profile with predominantly mild gastrointestinal effects and rare liver enzyme elevations [15,16]. Recent meta-analyses further confirm its tolerability and consistent efficacy over treatment durations ranging from 36 to 52 weeks [17].

In parallel with its regulatory approval, a multidisciplinary expert panel developed clinical guidance in 2024 to support healthcare providers in identifying suitable patients and implementing monitoring protocols for Resmetirom in NASH cases with F2-F3 fibrosis [18]. Comparative analyses have also placed Resmetirom ahead of several pipeline agents, including fibroblast growth factor 21 (FGF21) analogs and glucagon-like peptide-1 (GLP‑1) receptor agonists, in terms of efficacy and safety outcomes [19].

Since its launch in the United States, preliminary real-world evidence has begun to emerge, supporting its tolerability and effectiveness in broader clinical settings, while also identifying some challenges related to access and reimbursement [20]. In Europe, the European Medicines Agency's (EMA) Committee for Medicinal Products for Human Use (CHMP) gave it a positive opinion in June 2025, with plans for a broader rollout by the end of the year [21].

Given its status as the first FDA-approved therapy for MASH with moderate to advanced fibrosis, Resmetirom represents a paradigm shift. However, with limited global rollout, uncertainties persist around real-world utilization, equity, safety in special populations, and long-term impact. 

This scoping review aims to: (1) synthesize real-world data on Resmetirom’s efficacy and safety; (2) explore implementation factors including diagnostic strategies, cost, and access; and (3) identify evidence gaps to inform future research and equitable practice.

## Review

Method

This review followed a structured scoping approach guided by the PRISMA-ScR (Preferred Reporting Items for Systematic Reviews and Meta-Analyses extension for Scoping Reviews) Checklist [22], aiming to assess post-approval evidence on the use of Resmetirom for treating noncirrhotic NASH with moderate to advanced fibrosis (F2-F3) since its FDA approval in March 2024.

Search Strategy and Eligibility Criteria

A comprehensive literature search was conducted using PubMed, Google Scholar, and ClinicalTrials.gov, covering the period from April 2024 to May 2025. Additional sources included FDA and EMA regulatory portals, and conference abstracts such as those of the American Association for the Study of Liver Diseases (AASLD) and European Association for the Study of the Liver (EASL).

Table 1 outlines the PICOS (Population, Intervention, Comparison, Outcomes, and Study Designs) criteria used to guide the inclusion and exclusion of studies in this review. 

**Table 1:** PICOS Framework Defining the Inclusion and Exclusion Criteria for Studies Evaluating Resmetirom in MASH/NASH Patients MASH: Metabolic dysfunction associated steatohepatitis; NASH: nonalcoholic steatohepatitis; NITs: noninvasive tests; PICOS: population, intervention, comparison, outcomes, study designs; RCTs: randomized controlled trials; RWE: real-world evidence "–” indicates not applicable.

PICOS	Inclusion Criteria	Exclusion Criteria
Population	Adults with noncirrhotic MASH/NASH and F2-F3 Fibrosis	Adults with cirrhotic MASH/NASH and F0–F1 fibrosis (i.e., mild or no fibrosis)
		Children and Adolescents <18 years
		Population with multiple or complex health conditions
		Pregnant and lactating women
Intervention	Treatment with Resmetirom	Patients who do not receive Resmetirom treatment
	NITs for assessing eligibility for Resmetirom	Use of other thyroid hormone receptor agonists
Comparison	Placebo	Other treatments or active drugs, apart from placebo and standard of care
	Standard of Care	
	Diagnostics for treatment eligibility	
	Access and Cost	
Outcomes	Clinical efficacy and outcomes	Studies focusing solely on non-relevant outcomes (e.g., non-liver-related outcomes)
	Patient access	Studies that do not report on the key outcomes (e.g., clinical efficacy, diagnostic performance, cost-effectiveness)
	Diagnostic performance	-
	Cost-effectiveness	-
Study Designs	RCTs	Phase 1 to 3 Clinical Trials
	Observational Studies	Case reports
	RWE	Studies without control groups
	Reviews (systematic and others)	-
	Post-market surveillance reports	-
	Clinical trials (Phase IV)	-
	Other regulatory reports	-

The search strategy included keywords such as “resmetirom” AND “NASH” AND “MASH” AND “cost” AND “long-term effects” AND “real-world” AND “accessibility” AND “marketing” AND “implementation outcomes,” AND “health economics” on PubMed, with filters applied to exclude Clinical Trial Phase I, Phase II, and Phase III studies. On Google Scholar, the keywords used were “resmetirom” AND “NASH” AND “MASH” AND “marketing” AND “real-world” AND “cost.

Data Extraction and Synthesis

In line with PRISMA 2020 guidelines [23], data extraction was independently conducted by two reviewers using a standardized template aligned with the PICOS framework. Inclusion was determined based on predefined PICOS criteria. Due to the variability in study design, outcome measures, and follow-up periods, a qualitative synthesis approach was applied. Any discrepancies in data extraction were resolved through discussion and consensus.

Only English-language, full-text studies were included, while conference abstracts were reviewed at the summary level without full evaluation. Although pivotal trials that led to Resmetirom’s regulatory approval were not reassessed, their findings were referenced in the Results section to benchmark and contextualize real-world outcomes.

The data were managed using Microsoft Excel (Microsoft Corp., Redmond, WA, USA). Duplicate records were identified and removed using built-in Excel functions to ensure accuracy and consistency. Findings were summarized narratively, mapped, and organized into key thematic categories to support interpretation and highlight patterns across the studies.

Managing the Risk of Bias

Given the diverse nature of the studies included, which didn't align with a single Risk of Bias (RoB) tool, no specific RoB tool was used in this review, as it is not mandated by the PRISMA-ScR [22] guidelines. However, several steps were taken to minimize potential bias.

Data extraction was performed independently by the two authors based on the PICOS framework, ensuring consistency and reducing the likelihood of selection bias. Any disagreements were resolved through discussion and consensus to ensure reliability. Studies were selected based on predefined criteria, helping to keep the process objective and systematic. Both authors independently screened all studies to ensure a comprehensive and unbiased selection.

The quality of studies was evaluated based on factors like sample size, study design, and outcome measures, with any studies identified as high-risk for bias (e.g., lacking control groups or relying on noninvasive methods without biopsy confirmation) noted in the limitations section. Efforts were also made to minimize reporting bias by ensuring consistent reporting of key data. These measures aimed to reduce bias and ensure the findings are based on reliable and systematically evaluated evidence.

Results

**Figure 1:**
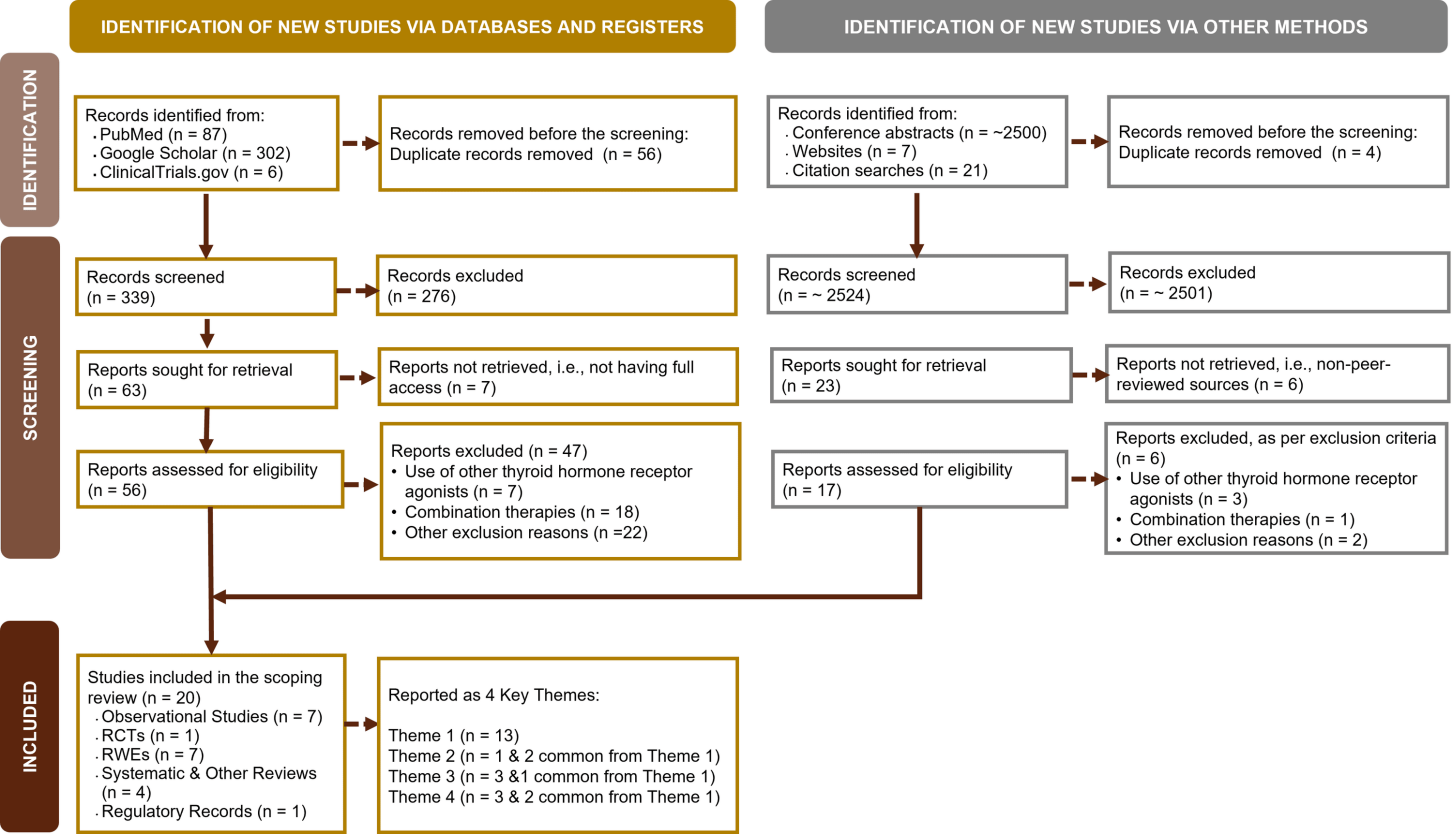
PRISMA 2020 Flow Diagram Depicting the Study Selection Process PRISMA: Preferred Reporting Items for Systematic Reviews and Meta-Analyses; RCTs: randomized clinical trials; RWEs: real-world evidences

In alignment with PRISMA 2020 guidelines [23], the study selection process is illustrated in Figure 1. After removing duplicates and screening titles, abstracts, and full-text articles, eligible studies were included based on predefined PICOS criteria. A total of 20 studies met the inclusion criteria and were analyzed.

The following findings integrates evidence from pivotal trials and emerging real-world data to evaluate Resmetirom's role in treating MASH with fibrosis, organized into four key domains: (1) clinical effectiveness, diagnostics, and safety; (2) patient experience and quality of life; (3) cost and health economics; and (4) implementation and access barriers.

Clinical Effectiveness, Diagnostics, and Safety

Phase III clinical trials and subsequent expert analyses have consistently demonstrated that Resmetirom significantly reduces hepatic fat content, promotes MASH resolution, and improves fibrosis scores, particularly in patients with biopsy-confirmed F2 or F3 fibrosis [18,24,25].

Real-world practice has increasingly shifted from biopsy to noninvasive tests (NITs), including FibroScan, controlled attenuation parameter (CAP), vibration-controlled transient elastography (VCTE), and enhanced liver fibrosis (ELF) panels, to determine patient eligibility and monitor treatment response [24,26-28].

However, emerging evidence suggests that reliance on traditional algorithms like the Fibrosis-4 (FIB-4) index, as recommended by AASLD and EASL, may fail to identify nearly half of patients with significant fibrosis [29]. In contrast, patients who meet AASLD criteria for Resmetirom initiation, particularly those with liver stiffness measurements (LSM) below 10 kPa, tend to experience fewer liver-related events [30]. Meanwhile, novel scoring systems such as Simplified Acute Physiologic Score (SAFE) and the mAchine Learning ADvanceD fibrosis and rIsk metabolic dysfunction-associated steatohepatitis Novel predictor (ALADDIN)-F2-Lab, which utilize standard laboratory markers, more accurately classify patients into intermediate or high-risk categories. These findings support the need for confirmatory testing and more refined diagnostic strategies to ensure timely identification and treatment of MASH [31].

In a smaller real-world analysis, the first 19 patients treated with Resmetirom showed reductions in liver stiffness by the three-month mark, independent of weight loss, glycemic control, or use of GLP-1 receptor agonists. These early improvements suggest the potential utility of short-term response assessment in high-risk patients, though the findings are limited by the absence of a control group and short follow-up duration [32].

A larger real-world cohort reinforced the high cardiometabolic burden in MASH patients receiving Resmetirom. Most had advanced fibrosis (F2-F3) diagnosed via transient elastography, along with prevalent comorbidities such as obesity and type 2 diabetes. These observations underscore the need for integrated cardiometabolic care and validate the role of Resmetirom as a therapeutic cornerstone in MASH management [33].

Across both clinical trials and real-world studies, Resmetirom has shown a favorable safety profile. Adverse events were typically mild, with gastrointestinal symptoms and pruritus being most common, and overall tolerability comparable to placebo [25].

Real-world data support these findings. At two tertiary care centers, the majority of patients receiving Resmetirom had advanced fibrosis confirmed by NITs, with a median age of 56 years, and commonly received co-prescriptions of GLP-1 agonists and statins, highlighting its relevance and applicability in real-world clinical settings [34]. In another multicenter study, limited to a geographic location, 97% of 113 patients were approved following insurance review, though 16% discontinued due to side effects such as nausea, diarrhea, or rash [20]. A separate retrospective review showed that 85.5% of patients completed at least three months of therapy, with an 8.8% discontinuation rate and modest improvements in liver enzymes [35].

Despite these favorable outcomes, Resmetirom’s safety and efficacy remain unconfirmed in special populations, including pediatric patients, pregnant or breastfeeding women, those with severe renal impairment, and individuals with NASH-related cirrhosis [36]. Since older adults (≥65 years) appear to experience a higher incidence of adverse reactions, highlighting the need for age-specific monitoring protocols.

Patient Experience and Quality of Life

While initial trials of Resmetirom emphasized histologic endpoints, emerging real-world data point to benefits in workflow efficiency, care continuity, and access. A multistakeholder prescribing model integrating hepatology providers, pharmacists, and insurance liaisons improved timely access and reduced care fragmentation, replacing the outdated “prescribe and forget” approach with a structured pathway involving regular monitoring and clinical engagement [20].

Patient populations treated with Resmetirom in practice resemble the broader MASH demographic, predominantly middle-aged individuals with obesity and type 2 diabetes, suggesting the potential for a scalable health system impact if access is expanded [24]. Although direct quality-of-life (QoL) metrics are limited in real-world settings, the overall patient experience appears favorable (i.e., 85.5% completed at least three months of treatment, nausea, vomiting, and diarrhea in 12.4%), as reflected by high treatment completion rates and manageable side effects [35].

In controlled trials, improvements in health-related quality of life (HRQoL) have been formally demonstrated. A 52-week randomized trial found that patients on Resmetirom experienced significant gains in emotional well-being, reduced stigma, and alleviated disease-related worry, measured through validated instruments like the Chronic Liver Disease Questionnaire - Non-alcoholic Fatty Liver Disease (CLDQ-NAFLD) and Liver Disease Quality of Life (LDQOL) [37]. These improvements were particularly marked in histologic responders, reinforcing the relevance of HRQoL as a complementary therapeutic goal [25,37].

Cost and Health Economics

Despite its clinical efficacy, Resmetirom’s high cost presents a major barrier to widespread adoption. A value-based pricing analysis estimated the incremental cost-effectiveness ratio (ICER) at $140,134 per quality-adjusted life year (QALY) gained compared to standard of care (SoC), exceeding the commonly accepted thresholds of $50,000-$100,000 per QALY [38]. Although, compared with SoC, the drug added 0.26 QALYs per patient and reduced liver-related medical costs by approximately $18,499 in comparison to a total incremental cost, the steep lifetime drug price of $54,754 per patient limited overall cost-effectiveness [38].

Interestingly, sensitivity analyses revealed that lower discontinuation rates, though clinically desirable, could increase cumulative costs and further reduce cost-effectiveness [38]. In real-world practice, however, moderate discontinuation rates and common delays due to insurance pre-authorization may partly mitigate this financial impact [20,35].

Further studies suggest that, in its current form, Resmetirom is not cost-effective at a $100,000/QALY willingness-to-pay threshold [38]. Budget impact models indicate a moderate rise in net budget impact of $2.2-$9.5 million with per-member per-month costs ($0.19-$0.80) for private insurers, especially in the early adoption phase [39]. Overall, cost-effectiveness remains highly sensitive to pricing, discontinuation rates, and long-term real-world outcomes, emphasizing the need for negotiated pricing frameworks and risk-based treatment stratification [40].

Implementation and Access Barriers

System readiness and equitable access remain pivotal for the successful implementation of Resmetirom. Real-world data show that accessing the drug often involves navigating a multistep process, including specialty pharmacy enrollment and insurance preauthorization, leading to prescription delays averaging 30 days [20]. In another analysis, 24% of patients required appeals to secure insurance approval, with average delays of 12.5 days before dispensing [35].

A major system-level advancement is the shift toward noninvasive diagnostic tools such as FibroScan and CAP in lieu of liver biopsy, which were widely used to assess eligibility in real-world prescribing cohorts [24]. The recommended VCTE thresholds successfully excluded most MASLD patients with early-stage (F0/F1) fibrosis from Resmetirom treatment. However, for those with VCTE values between 10 and 19.9 kPa and no signs of cirrhosis, further diagnostic evaluation, such as liver biopsy or advanced noninvasive tests, may be necessary to reduce misclassification and ensure accurate patient selection [41]. These tools offer scalable, less burdensome alternatives, particularly important in expanding access to underserved populations.

Long-term success will require investment in post-marketing surveillance to monitor adverse events, refine patient selection with treatment focused on "principal risk", and assess the broader impact on comorbidities and disease progression [42]. In parallel, managed care organizations (MCOs) must facilitate early patient identification, support provider and patient education, and foster integrated care pathways [43]. These efforts are essential to avoid deepening health disparities and ensure Resmetirom's benefits reach those most at risk.

While existing clinical trial outcomes affirm Resmetirom’s efficacy and tolerability in managing MASH with fibrosis, emerging post-marketing data underscore critical real-world barriers, including treatment discontinuation, insurance-related delays, and variability in diagnostic and access pathways, that may influence its broader clinical impact and equitable implementation.

Table 2 summarizes each study included in the review, outlining its main features and findings to show how the evidence contributes to a broader understanding of Resmetirom’s use across different clinical settings.

**Table 2:** Summary of Studies Included in this Review AASLD: American Association for the Study of Liver Diseases; BMI: body mass index; CAP: controlled attenuation parameter; ELF: enhanced liver fibrosis; FIB-4: fibrosis-4; GLP-1 RAs: Glucagon-like peptide 1 receptor agonists; HRQL: health-related quality of life; LFAST: liver fibroscan aspartate aminotransferase; LRE: liver-related events; LSM: liver stiffness measurement; MASLD: metabolic dysfunction associated steatotic liver disease; MASH: metabolic dysfunction-associated steatohepatitis; MCOs: managed care organizations; MRE: magnetic resonance elastography; NA: not applicable/not available; NASH: non alcoholic steatohepatitis; NITs: non-invasive tests; PMPM: per member per month; PR: progression rate; QALY: quality-adjusted life year; RCTs: randomized controlled trials; RWEs: real-world evidences; SAFE: steatosis-associated fibrosis estimator; T2D: type 2 diabetes; TE: transient elastography; VCTE: vibration-controlled transient elastography “–” indicates data not available, not applicable, or not reported.

Study/Source	Study Type	Year of Publication	Study Country	Population of Study	Aim of Study	Main Findings
Clinical Effectiveness, Diagnostics, and Safety						
Kaya et al., 2025 [24]	Clinical Review	2025	-	-	To summarize clinical insights on Resmetirom, including clinical indications, patient selection, and monitoring therapy response	Initiating therapy with Resmetirom does not require a liver biopsy.
						Accurate exclusion of patients with mild liver histology or cirrhosis is essential and should be achieved through NITs.
						Ongoing monitoring of treatment response should also be conducted using NITs.
						Clear discontinuation criteria are needed to prevent unnecessary treatment interruptions.
Bittla et al., 2024 [25]	Systemic Review	2024	-	-	To assess safety, efficacy, and other practical aspects of Resmetirom in the treatment of NASH	Resmetirom shows strong potential for NASH treatment, with benefits in cost-effectiveness, QALY, cardiovascular risk reduction, and patient-reported outcomes.
						Further research is needed across diverse populations to assess long-term effectiveness, comorbidities, fibrosis progression, and potential biases. Hence, Resmetirom remains investigational and is not yet an established therapy.
Schattenberg et al., 2025 [26]	Observational Study (Retrospective)	2025	-	1247 Patients with F0 to F4 on liver biopsy	To assess the use of NIT to diagnose and monitor NASH treated with Resmetirom	Effective identification of patients with NASH and fibrosis stages F2–F3 was achieved using NITs like FibroScan VCTE, ELF, and readily available blood tests.
						Expanding NIT criteria to include ELF and MRE may enhance accuracy in fibrosis staging.
Messer et al., 2025 [27]	RWE	2025	Germany	1113 patients across eight tertiary and 12 secondary care centers	To assess the eligibility for Resmetirom involving both tertiary and secondary care centers	Eligibility for Resmetirom depends on the method used to identify "at-risk MASH," with VCTE being the most widely available tool across levels of care.
Sandhu et al., 2025 [28]	Observational Study (Retrospective)	2025	NA	24 patients without Resmetirom discontinuation	To retrospectively assess the dynamics of NITs in initiating and monitoring patients on Resmetirom therapy, focusing on fibrosis, disease activity, steatosis PR, and mean change from baseline.	Initiation of Resmetirom based on NITs such as LFAST, FIB-4 (for fibrosis), and VCTE is effective and supports continued non-invasive monitoring. A rapid improvement in steatosis was observed.
Olveira et al., 2025 [29]	Observational Study (Cross-Sectional)	2025	NA	1281 patients with liver biopsy, comorbidity assessment, analytical profile, TE, and CAP.	To evaluate the validity of the proposed criteria for initiating Resmetirom treatment in patients with fibrotic MASH in real-world clinical practice.	Proposed non-invasive criteria were suboptimal, potentially excluding over half of eligible fibrotic MASH patients from treatment.
Yeow et al., 2025 [30]	Observational Study (Retrospective)	2025	NA	213 patients meeting LSM and Resmetirom indication criteria	To evaluate rates of LRE, non-liver-related deaths, and changes in liver stiffness (progression/regression) among MASLD patients meeting AASLD LSM criteria for Resmetirom.	LREs were rare in patients meeting AASLD criteria for Resmetirom, especially with LSM <10 kPa. Non-liver-related mortality was more common. Most patients showed LSM improvement without specific therapy, suggesting a high number needed to treat to prevent one medium-term LRE.
Dunn et al., 2025 [31]	RWE	2025	U.S.	424 patients on Resmetirom	To evaluate the performance of proposed noninvasive diagnostic pathways for risk-stratifying MASLD patients in primary care and identifying those needing hepatology referral for treatment consideration	Current AASLD and EASL guidelines relying on FIB-4 may miss nearly half of MASH patients with significant fibrosis.
						Novel scores like SAFE and ALADDIN-F2-Lab identified more at-risk patients using routine labs.
						Findings support the need for confirmatory testing and improved diagnostic strategies to enhance timely identification and treatment.
Saggese et al., 2025 [32]	RWE	2025	U.S.	36 patients on Resmetirom	To assess whether treatment response to Resmetirom can be effectively evaluated at an early 3-month timespan.	In the first 19 patients treated with Resmetirom, LSM decreased by three months, independent of liver enzymes, weight/BMI changes, diabetes control, or GLP-1 RA use.
Alkhouri et al., 2025 [33]	RWE	2025	U.S.	424 patients on Resmetirom	To describe the general characteristics of MASH patients prescribed Resmetirom, including noninvasive test results and concomitant medications.	MASH patients prescribed Resmetirom showed a high burden of cardiometabolic comorbidities, particularly obesity and T2D.
						Most patients were diagnosed with advanced fibrosis (F2-F3) using NITs like TE, marking a shift from biopsy reliance in clinical trials to real-world practice.
Kaya et al., 2025 [34]	RWE	2025	U.S.	72 patients on Resimetrom	To describe the general characteristics and NITs of MASH patients prescribed Resmetirom	LSM values varied widely, with only 46% falling within the 10–20 kPa range.
Shuaibi et al., 2025 [35]	RWE	2025	U.S.	137 patients on Resmetirom	To evaluate the tolerability of Resmetirom in treating MASH fibrosis and identify factors affecting access, including insurance coverage and dispensing delays, for patients who received at least three months of therapy.	Resmetirom is safe and well-tolerated for the first three months.
						Most patients gained insurance approval, though dispensing delays and requirements for invasive fibrosis testing were observed.
FDA Database, 2024 [36]	Regulatory Records	2024	-	-	Resmetirom information leaflet providing clear prescribing information for healthcare professionals.	Safety and efficacy have not been evaluated in special populations, including pediatric patients, pregnant or breastfeeding women
Patient Experience and Quality of Life						
Younossi et al., 2025 [37]	RCT	2025	U.S.	966 intention-to-treat patients: 323 received Resmetirom 100 mg, 322 Resmetirom 80 mg, and 321 placebo	To assess HRQL in patients with MASH treated with Resmetirom.	Resmetirom led to significant, meaningful HRQL improvements in patients with MASH/NASH showing fibrosis or disease resolution.
Cost and Health Economics						
Le et al., 2025 [38]	Observational Study	2025	-	132,600 patients with MASH-F3	To assess the lifetime cost-effectiveness of Resmetirom vs. standard care and identify its cost-effective price threshold.	At $19,011/year, Resmetirom was not cost-effective versus standard care at a $100,000/QALY threshold, with results sensitive to discontinuation rates and model assumptions.
Fishman et al., 2024 [39]	Observational Study	2024	-	-	To assess the budget impact and total projected costs of Resmetirom for treating non-cirrhotic NASH with moderate-to-advanced fibrosis	Resmetirom's introduction led to 50–238 treated patients and a net budget impact of $2.2-$9.5 million, with PMPM costs of $0.19-$0.80 over years 1-3.
Lazarus, 2025 [40]	Review (Commentary)	2025	-	-	To evaluate the value of treating MASH in terms of clinical and economic outcomes.	Cost-effectiveness is highly sensitive to pricing, discontinuation rates, and long-term outcomes, highlighting the need for negotiated pricing and risk-based treatment strategies.
Implementation and Access Barriers						
John et al., 2025 [41]	RWE	2025	Germany	291 for VCTE	To assess the eligibility of Resmetirom based on NITs	VCTE <10 kPa indicated low fibrosis, while ≥20 kPa signaled advanced fibrosis or cirrhosis. Many patients with VCTE 10-19.9 kPa did not meet Resmetirom criteria despite lacking cirrhosis features. Narrowing the range to 10-15 kPa reduced off-label classifications but remained suboptimal.
Petroni et al., 2024 [42]	Review	2024	-	-	To trace the history of treatment and explore how new achievements are shaping future practices.	MASLD should be initially screened by general practitioners using surrogate biomarkers. Patients with NASH, fibrosis, or comorbidities should be referred to a multidisciplinary team, with treatment focused on "principal risk" and liver as a target only if fibrosis is present, alongside promoting healthier lifestyles.
Flavin, 2024 [43]	Observational Study	2024	-	-	To propose managed care for early intervention, ensure equitable access, and integrate the patient perspective in the emerging market of NASH/MASH.	With rising NASH/MASH prevalence and emerging treatments, MCOs must support equitable care, promote early diagnosis, and enhance access to personalized care, while exploring strategies for new treatments.

Discussion

The liver and thyroid interact closely to maintain metabolic balance. Thyroid disorders affect hepatic glucose and lipid metabolism, contributing to conditions like hypercholesterolemia and MASLD. Conversely, liver dysfunction can impair thyroid hormone levels and their action on peripheral tissues [44].

MASLD affects over 30% of the global population, making it one of the most prevalent liver conditions worldwide. The health and economic burdens are significant, particularly in the United States, where MASLD contributes to over $100 billion in healthcare spending annually. The burden is even more pronounced in regions like the Middle East and North Africa, where high rates of type 2 diabetes, largely driven by insulin resistance, have fueled a surge in MASLD cases. Notably, approximately 65% of individuals with type 2 diabetes also develop MASH, underscoring the shared pathophysiology between these conditions [45,46].

The global prevalence of MASLD is projected to rise from 38.9% in 2020 to 55.7% by 2040, a 43.2% increase [47], highlighting a growing public health concern and the urgent need for effective prevention and treatment strategies.

The FDA’s approval of Resmetirom in 2024 marked a pivotal milestone as the first medication specifically approved for adults with noncirrhotic MASH and moderate to advanced fibrosis [10]. Available in oral tablet form at 60 mg, 80 mg, and 100 mg, it offers a practical and scalable therapeutic option following decades of reliance on lifestyle modifications alone. However, several questions remain regarding its real-world effectiveness, safety across diverse populations, cost-effectiveness, and overall implementation.

To address these uncertainties, the FDA has required the manufacturer to complete a confirmatory post-marketing study (Trial MGL-3196-11) by August 2028 [48]. This trial aims to generate important data on long-term outcomes, such as progression to cirrhosis, hepatic decompensation events like liver failure, the need for transplant, and mortality. These results will be essential to confirm whether the benefits seen in trials translate into real-life clinical impact such as reductions in cirrhosis incidence and all-cause mortality.

Clinical Outcomes and Safety Signals

Resmetirom has demonstrated a generally favorable safety profile across clinical trials, with most adverse events reported as mild to moderate in severity [3]. The most commonly observed side effects were gastrointestinal in nature, primarily diarrhea and nausea, which occurred more frequently in patients receiving Resmetirom compared to those on placebo. Importantly, these symptoms were typically short-lived and manageable, rarely requiring treatment discontinuation [17].

A key factor contributing to Resmetirom’s safety is its selective activity on thyroid hormone receptor beta (THR-β), which is predominantly expressed in the liver. This targeted mechanism allows Resmetirom to modulate liver metabolism while minimizing effects on systemic thyroid hormone function. Although reductions in free thyroxine (FT4) levels of approximately 16%-19% have been observed, levels of free triiodothyronine (FT3) and thyrotropin (TSH) remain largely unchanged, thereby reducing the risk of systemic hypothyroidism [15]. By avoiding activation of thyroid hormone receptor alpha (THR-α), which is more widely distributed in tissues such as the heart and bones, Resmetirom also helps minimize off-target effects, such as cardiovascular or skeletal complications [49].

Although no cases of severe liver injury have been reported, rare, asymptomatic elevations in liver enzymes have occurred, underscoring the need for routine liver function monitoring, especially in patients with existing liver conditions or those on potentially hepatotoxic drugs [42,50]. Upon treatment for up to two years, Resmetirom has shown improvements in liver stiffness, fibrosis biomarkers, fibrosis scores, and portal hypertension risk in patients with compensated MASH cirrhosis [51].

Resmetirom has also been identified through a multimodal AI model as having a favorable safety profile among therapies for MASH. By integrating diverse preclinical data, the model supports its potential for safe use in polypharmacy and complex metabolic conditions [52].

Real-world pharmacovigilance data support these findings. As of March 31, 2025, the U.S. FDA Adverse Event Reporting System (FAERS) had recorded 390 adverse events associated with Resmetirom, most of which were gastrointestinal in nature and consistent with clinical trial data [20,53-54].

Generalizability and Real-World Relevance

While the results from clinical trials have been promising, how well they apply to everyday clinical practice is still uncertain. Most of the early studies after Resmetirom’s approval were done in large, tertiary care settings in the United States, where patients often have access to advanced tests and close follow-up. This isn't always the case in smaller clinics or in countries with fewer resources. Also, many of the real-world studies so far have included only a small number of patients or had short follow-up periods, which makes it harder to know how Resmetirom will perform for the broader population in the long run. Therefore, it is important that real-world studies also be conducted in non-tertiary settings, such as community clinics and primary care populations, to support broader generalizability.

Moreover, the pivotal trials that led to Resmetirom’s approval excluded important patient groups, such as children and adolescents under 18, older adults, individuals with decompensated cirrhosis, and those with multiple or complex health conditions. This limits how well the trial results apply to real-world populations and leaves significant gaps in our understanding of how safe and effective Resmetirom is for these patients.

Risk of Bias and Limitations in Post-Marketing Data

Post-marketing studies, particularly those sponsored by industry, carry inherent risks of bias. Selection bias may arise as patients prescribed Resmetirom tend to be more engaged, insured, or referred to tertiary centers. In addition, reliance on surrogate markers such as alanine transaminase (ALT) or low-density lipoprotein cholesterol (LDL-C) without histological confirmation may overstate effectiveness [55,56].

While early real-world registries are conducted under ethical oversight, the lack of long-term independent datasets is a notable limitation. Broader participation across diverse, multicenter settings with transparent reporting will be essential for generating robust, generalizable data [57].

Access and Equity Considerations

Resmetirom is now commercially available in the United States and has received conditional marketing authorization from the EMA. In the European Union (EU), access will vary by country, with Germany expected to launch the product in late 2025 [10,58]. In the United Arab Emirates (UAE), Resmetirom is already available [59], presenting an important opportunity to accelerate regional real-world data generation. Given the high burden of MASLD and type 2 diabetes in the Middle East [60], early local uptake can provide valuable insights into access, adherence, and treatment outcomes in diverse patient populations, especially in non-Western settings where such data are limited.

Early prescribing patterns indicate a focus on hepatology and gastroenterology practices [61], which may inadvertently delay access for underserved or community-based populations. Without coordinated public health strategies, including policy reforms, targeted awareness campaigns, and expanded screening, there is a risk that those most affected by MASH, particularly in underserved populations, may not benefit from this therapeutic advancement in the near future [61-63].

Furthermore, Resmetirom has not been studied in certain high-need groups, including patients with cirrhosis, children, and women who are pregnant, breastfeeding, or planning pregnancy, limiting its use in these populations and raising concerns about equity in liver care [61]. Consequently, adaptive or seamless Phase 2/3 trial designs incorporating Bayesian monitoring are urgently needed to generate critical safety and efficacy data and to effectively assess Resmetirom’s performance in these underrepresented populations.

A recent study highlights the rising global prevalence of pediatric MASLD, its association with significant childhood comorbidities, and a markedly increased risk of early adult mortality. Key barriers include limited access to care, insufficient lifestyle support programs, lack of multidisciplinary approaches, minimal community engagement, and a need for age-specific clinical research [64].

Hence, without targeted research efforts, these populations are often excluded from emerging advancements, thereby perpetuating existing healthcare disparities.

Affordability and Health System Readiness

At a cost of $19,011 per year in the United States, Resmetirom was not considered cost-effective compared to standard-of-care when evaluated against the $100,000/QALY threshold [38]. Physicians have also expressed concerns over affordability and insurance-related delays, which may further impact uptake and long-term adherence [65].

To support access, Madrigal Pharmaceuticals offers a Patient Support Program, which provides insurance navigation, co-pay assistance, and a Patient Assistance Program (PAP) for the uninsured [66]. However, broader payer coverage, shown to enhance access and streamline reimbursement in comparator conditions such as cardiovascular diseases and diabetes, will be essential to ensure equitable access.

In March 2025, AASLD issued a position statement advocating for coverage models that prioritize both disease burden and equity [67]. Globally, the EMA’s CHMP granted a positive opinion for conditional approval in June 2025, based on bridging Phase 3 data and unmet clinical need [58]. Nevertheless, delayed launches in Europe and other regions are expected due to ongoing health technology assessments (HTAs) and pricing negotiations [68].

Population Modeling and Future Research

While the MAESTRO-NAFLD Open-Label Extension (MAESTRO-NAFLD-OLE) trial is actively enrolling participants across 74 sites in the United States and Puerto Rico to further assess the safety and biomarker response to Resmetirom [69], real-world projections remain concerning. Current estimates suggest that less than 15% of NASH cases in the United States will be diagnosed within the first three years of Resmetirom's regulatory approval, greatly limiting the number of patients who could benefit from the treatment [70]. This highlights the urgent need to strengthen screening efforts, particularly in primary care settings, to improve early detection and timely management across the disease spectrum.

As such, future research must focus on expanding eligibility through inclusive trial designs, long-term safety monitoring in special populations, and evaluating combination therapies or treatment discontinuation strategies. Crucially, studies are needed to determine whether the histological and metabolic improvements achieved with Resmetirom are maintained after therapy cessation or if NASH relapse occurs, an important yet currently unknown parameter.

Understanding the durability of response will be essential in guiding maintenance strategies, assessing the need for retreatment, and defining long-term treatment goals. Rational prescribing frameworks, particularly in the context of polypharmacy and multimorbidity, will also be critical to ensuring personalized, sustainable care.

Review Limitations

The available data are still early and predominantly focused on studies from the United States, involving small patient cohorts, short follow-up durations, and limited representation of global or high-risk populations. Many of the studies lacked control groups and relied on noninvasive diagnostic methods without histologic confirmation, which may have exaggerated the perceived treatment effects. Additionally, the included studies mainly featured early users of Resmetirom who were more likely to be insured and treated in specialized or tertiary care settings, leading to an underrepresentation of patients paying out-of-pocket or those managed under managed care organizations (MCOs).

Furthermore, since this review was limited to the first year of Resmetirom’s post-approval data, it did not explore its effectiveness or safety in combination with other medications such as GLP-1 agonists, an area that warrants further investigation. Lastly, the review was restricted to English-language publications from April 2024 to May 2025 due to constraints in translating and interpreting non-English studies, which may have excluded relevant studies published in other languages or outside this timeframe. 

Additionally, data management was conducted using Excel rather than advanced tools like Covidence or Rayyan, due to limited resource availability, potentially limiting the depth of data handling.

## Conclusions

In conclusion, Resmetirom’s approval marks a pivotal step in treating noncirrhotic MASH with moderate to advanced fibrosis, offering a long-awaited alternative to lifestyle-only approaches. However, its real-world effectiveness is still uncertain due to short follow-up periods in studies, limited data from diverse populations, low diagnosis rates, unequal access, high costs, and the exclusion of important patient groups from trials.

To realize its full potential, efforts should focus on adopting health system strategies such as multidisciplinary care models, policy-level initiatives (e.g., value-based pricing, public-private partnerships) to enhance access, expanding screening programs, ensuring equity, addressing affordability, and generating long-term, inclusive safety and efficacy data. Ultimately, the success of Resmetirom will depend not only on its clinical benefits but also on how effectively health systems integrate it into comprehensive, patient-centered liver care.
